# Tin-Decorated Reduced Graphene Oxide and NaLi_0.2_Ni_0.25_Mn_0.75_O_δ_ as Electrode Materials for Sodium-Ion Batteries

**DOI:** 10.3390/ma12071074

**Published:** 2019-04-01

**Authors:** Pier Paolo Prosini, Maria Carewska, Cinzia Cento, Gabriele Tarquini, Fabio Maroni, Agnese Birrozzi, Francesco Nobili

**Affiliations:** 1 ENEA, Italian National Agency for New Technologies, Energy and Sustainable Economic Development, Casaccia Research Centre, Via Anguillarese 301, Santa Maria di Galeria, 00123 Rome, Italy; maria.carewska@enea.it (M.C.); cinzia.cento@enea.it (C.C.); 2 Dip. Scienze di Base ed Applicate per l’Ingegneria, University of Roma “La Sapienza”, Via Scarpa 14, 00161 Roma, Italy; gabriele.tarquini@uniroma.it; 3 School of Science and Technology, Chemistry Division, University of Camerino, Via S. Agostino, 62032 Camerino, Italy; fabio.maroni@unicam.it (F.M.); agnese.birrozzi@unicam.it (A.B.); francesco.nobili@unicam.it (F.N.)

**Keywords:** tin, reduced graphene oxide, NaLi_0.2_Ni_0.25_Mn_0.75_O_δ_, composite electrodes, sodium-ion battery

## Abstract

A tin-decorated reduced graphene oxide, originally developed for lithium-ion batteries, has been investigated as an anode in sodium-ion batteries. The composite has been synthetized through microwave reduction of poly acrylic acid functionalized graphene oxide and a tin oxide organic precursor. The final product morphology reveals a composite in which Sn and SnO_2_ nanoparticles are homogenously distributed into the reduced graphene oxide matrix. The XRD confirms the initial simultaneous presence of Sn and SnO_2_ particles. SnRGO electrodes, prepared using Super-P carbon as conducting additive and Pattex PL50 as aqueous binder, were investigated in a sodium metal cell. The Sn-RGO showed a high irreversible first cycle capacity: only 52% of the first cycle discharge capacity was recovered in the following charge cycle. After three cycles, a stable SEI layer was developed and the cell began to work reversibly: the practical reversible capability of the material was 170 mA·h·g^−1^. Subsequently, a material of formula NaLi_0.2_Ni_0.25_Mn_0.75_O_δ_ was synthesized by solid-state chemistry. It was found that the cathode showed a high degree of crystallization with hexagonal P2-structure, space group P6_3_/mmc. The material was electrochemically characterized in sodium cell: the discharge-specific capacity increased with cycling, reaching at the end of the fifth cycle a capacity of 82 mA·h·g^−1^. After testing as a secondary cathode in a sodium metal cell, NaLi_0.2_Ni_0.25_Mn_0.75_O_δ_ was coupled with SnRGO anode to form a sodium-ion cell. The electrochemical characterization allowed confirmation that the battery was able to reversibly cycle sodium ions. The cell’s power response was evaluated by discharging the SIB at different rates. At the lower discharge rate, the anode capacity approached the rated value (170 mA·h·g^−1^). By increasing the discharge current, the capacity decreased but the decline was not so pronounced: the anode discharged about 80% of the rated capacity at 1 C rate and more than 50% at 5 C rate.

## 1. Introduction

Lithium-ion batteries (LIBs) represent, without any doubt, the more efficient electrochemical technology for storing electricity. However, their development for large-scale applications is limited by the fact that lithium is not so abundant in the Earth’s crust. In fact, although lithium is found in many rocks and some brines, it is a rare element: lithium appears only 33rd on the scale of the relative abundances of the elements, constituting only 0.002% of the Earth’s crust [1]. On the other hand, sodium is very abundant; the Earth’s crust contains 2.6% sodium by weight, making it the sixth most abundant element on the Earth. For this reason, rechargeable electrochemical cells based on sodium appear very promising for large-scale storage applications [2] and represent a valid alternative to replace the most widespread and costly lithium batteries. With respect to lithium, sodium shows a higher redox potential (E°_(Na+/Na)_ = −2.71 V versus standard hydrogen electrode; about 0.3 V above that of lithium) and this could result in a lower energy density. Sodium metal batteries were initially developed as high-temperature batteries [3]. More recently, research activities have been addressed to room-temperature-operating sodium-ion batteries (SIBs). Indeed, studies of intercalation materials suitable for use as cathodes in SIBs began around the 1980s [4,5], but the rapid development of lithium-ion batteries slowed down the research. In the last few years, a renewed interest in SIBs was observed and several materials have been extensively studied as suitable anodes and cathodes for SIB applications. Apart from the use of two different alkaline metals, the operating principles of SIBs and LIBs are practically identical and many of the electrode materials used in SIB technology have been borrowed from LIB technology. Among them, transition metal oxides of general formula Na_x_MeO_2_ (Me = transition metal) such as NaNiO_2_ and NaCrO_2_ [4], NaMnO_2_ [5], Na_x_CoO_2_ [6], Na_x_VO_2_ [7], NaFeO_2_ [8], Na_x_TiO_2_ [9], double metal oxides of general formula Na_x_Me^1^_y_Me^2^_z_O_4_ such as Na_x_Mo_2_O_4_ [10], NaLi_0.2_Ni_0.2_Mn_0.6_O_2_ [11], Na_(x)_[Fe_1/2_Mn_1/2_]O_2_ [12], polyanionic compounds of general formula Na_x_Me_y_(PO_4_)_z_F_w_ (Me = transition metal) such as Na_2_FePO_4_F/C [13], NaFePO_4_ [14], Na_3_V_2_(PO_4_)_3_ [15], and Na_3_V_2_(PO_4_)_2_F_3_ [16], and Prussian blue (NaFe^III^Fe^II^(CN)_6_) [17] and its analogues such as Na_x_MeFe(CN)_6_, with Me = Fe [18], Co [19], Ni [18], and Mn [20] have been investigated. On the other hand, graphite, the most commonly used anode in lithium ion cells, does not interact with sodium ions in an appreciable manner [21]. For this reason, the identification of a suitable negative electrode able to store sodium ions is the key to the success for the development of SIBs. Research on negative electrode materials for SIBs has been addressed in the following four different categories: (1) carbonaceous materials, (2) polyanionic compounds (such as phosphates), (3) oxides and sulfides, and (4) p-block elements (metal alloys) [22]. The research on carbonaceous materials able to reversibly intercalate sodium was focused on soft and hard carbons. A soft carbon, prepared by pyrolysis of petroleum cokes, has been tested as an anode for SIBs [23] and a good result was obtained when cycling the cells at relatively high temperature (86 °C). The electrochemical reversibility of Na insertion/extraction at room temperature into/from hard carbon was reported in 2000 [24]. The achieved reversible capacity was 300 mA·h·g^−1^, close to that usually obtained for lithium insertion in graphitic materials. In 2014, the reversible Na intercalation into graphite was demonstrated [25] in diglyme-based electrolyte. The cell exhibited 100 mA·h·g^−1^ of reversible capacity with excellent capacity retention. The second class of anode for SIBs was represented by polyanionic compounds. A typical representative of this class of compound was the NaTi_2_(PO_4_)_3_ [26]. Inside the class of metal oxides, particular attention was reserved for titanium oxides and their polymorphs. Just to cite one representative of this class, a nanosized anatase (with an average grain dimension lower than 30 nm) has been reported to be electrochemically active in Na cells [27]. The fourth class of compounds used as anodes in SIBs technology is the sodium alloys. Group 14 and 15 elements, including metals (Sn, Pb, Bi), metalloids (Si, Ge, As, Sb), and polyatomic nonmetal (P), were known to form binary compounds with Na. Among them, Sn [28] and Sb [29] and Sn–Sb alloy [30] have been extensively studied as potential negative electrodes in SIBs. It has also been demonstrated that the reversibility of the alloying/dealloying for Sn electrodes was improved by use of a polyacrylate (PAA) binder [31]. Inside this class, particular attention was deserved for material based on tin and reduced graphene oxide (RGO) such as: layered SnS_2_ [32], Sn/Sb alloys [33], SnSe nanoparticles [34], Sn_4_P_3_ [35], and SnO_2_ nanoparticles [36,37,38]. In a previous work [39], it was demonstrated that tin-decorated RGO (Sn-RGO) can be used as a negative electrode in LIBs. In this work, we tested the Sn-RGO as an anode for SIBs. First of all, the material was analyzed by XRD, TGA, and SEM. In agreement with the previous reported literature [31,39], a commercially available poly styrene-acrylate copolymer (Pattex PL50) was used as a binder to enhance the electrode characteristics of the Sn-RGO. The basic idea was that the Sn particles dispersed between the graphene sheets can reduce the irreversible aggregation of graphene layers to form graphitic stacks, allowing the storage of sodium ions through the chemisorption on heteroatoms and reversible surface physical adsorption on RGO. Furthermore, the Sn particle, by forming an alloy with sodium, can contribute to increasing the capacity of the material. A layered oxide was used as the cathode. According to Kim [40] et al., the following stoichiometry was chosen for the cathode active material: Na_1.0_Li_0.2_Ni_0.25_Mn_0.75_O_δ_. Lithium was introduced to stabilize the structure similarly to that observed in lithium-rich transition metal oxides such as the Li_1.2_Ni_0.2_Mn_0.6_O_2_ [41,42,43]. The material was characterized by XRD and SEM. An electrode was prepared by using Teflon as the binder and the electrochemical performance of the electrode was evaluated in a sodium cell. Finally, to assess the feasibility of the Sn-RGO as an anode in a full SIB, the Sn-RGO electrode was coupled with the Na_1.0_Li_0.2_Ni_0.25_Mn_0.75_O_δ_ electrode. The so-obtained SIB was cycled at various rates and its performance was evaluated by monitoring the capacity as a function of the discharge rate and cycle number.

## 2. Materials and Methods

### 2.1. Preparation of the Sn-Decorated RGO

The synthesis procedure was detailed elsewhere [44]. Briefly, 0.5 g of GO (Nanoinnova Technologies SL, Madrid, Spain) were dispersed by sonication in 50 mL of ethylene glycol (EG) until the formation of a stable suspension. A 1.50 g portion of polyacrylic acid (PAA, MW ~2000, Sigma Aldrich, Milano, Italy) was dispersed by sonication in about 20 mL of EG. A 0.05 M solution of phenyl tin chloride [(C_6_H_5_)_2_SnCl_2_] was prepared dissolving 0.344 g of the salt in about 20 mL of EG. The dispersion of PAA was added to the dispersion of GO dropwise and the resulting suspension was allowed to sonicate for two hours. The solution of phenyl tin chloride was slowly added dropwise to the suspension of GO/PAA under vigorous stirring. The resulting mixture was sonicated for 2 h. The reaction mixture was placed in a microwave oven and irradiated with a power of 900 W for 10 min under stirring. The black solid that formed was separated from the residual solution by filtration, washed three times with ethanol, and dried in air at 50 °C. The residue was heated in an oven at 800 °C under Ar/H_2_ atmosphere (95:5) for five hours and left to cool at room temperature.

### 2.2. Preparation of the Sn-Decorated RGO Anode Tape

A 0.6 g portion of Sn-decorated RGO and 0.136 g of carbon black (Super P, MMM Carbon, Brussels, Belgium) were weighed and transferred to a mill. Then, 0.064 g of Pattex PL50 (Henkel, Düsseldorf, Germany) with a solid mass fraction of 90% was dispersed in 10 g of water. The suspension was subsequently added to the powder mixture and the components were mixed by operating the mill for a few minutes. The so-obtained suspension was used to paint a copper sheet covering a surface area of 100 cm^2^. After drying in air at 130–150 °C, the procedure was repeated to use up the entire suspension. A typical negative electrode composition was 75.8 wt.% SnRGO, 7.0 wt.% Pattex PL50, and 17.2 wt.% Super P. Electrodes were cut in the form of a disc with a diameter of 12 mm and an average thickness of 80 μm. The electrode weight ranged from 4.8 to 5.2 mg, which corresponds to an active material specific mass loading of 3.22–3.48 mg·cm^−2^. Lighter electrodes (from 0.9 to 1.2 mg with a specific mass loading of 0.60–0.80 mg·cm^−2^) were obtained by punching the electrode tape in proximity of the edges. Prior to the electrochemical characterization, the electrodes were dried by heating under vacuum at 110 °C for 12 h.

### 2.3. Preparation of the Cathode Active Material

For the synthesis of the material, a variant of the acetate method as proposed by Kalapsazova et al. [45] was used. A 2.72 g portion of sodium acetate tri-hydrate (CH_3_COONa·3H_2_O, M.W.: 136.08, 0.02 mol, Aldrich), 0.408 g of lithium acetate bi-hydrate (CH_3_COOLi·2H_2_O, M.W.: 102.02, 0.04 mol, Aldrich), 1.244 g of nickel (II) acetate tetra-hydrate (Ni(OCOCH_3_)_2_·4H_2_O, M.W.: 248.84, 0.005 mol, Aldrich), and 3.676 g of manganese (II) acetate tetra-hydrate ((CH_3_COO)_2_Mn·4H_2_O, M.W.: 245.09, 0.015 mol, Aldrich) were loaded in a stainless steel vial, together with two stainless steel balls. The vial was mounted in a shaker mill (SPEX 8000, CertPrep, Metuchen, NJ, USA) and milled for 1 h. After milling, the mixture of acetates became liquid. The liquid was transferred to a ceramic crucible, heated in air at 800 °C for 4 h, and left to cool at room temperature.

### 2.4. Preparation of the Cathode Tape

Composite cathode tape was made by roll milling a mixture of 0.4 of active material, 0.0426 g of binder (Teflon, DuPont, Midland, MI, USA), and 0.0906 g carbon (Super P, MMM Carbon, Brussels, Belgium). The electrode tape composition was 75.0 wt.% active material, 8.0 wt.% Teflon, and 17 wt.% carbon Super P. Electrodes were punched from the tape in the form of discs with a diameter of 12 mm. The electrode weight ranged from 12.8 to 30.3 mg corresponding to an active material mass loading of 8.5–20.1 mg·cm^−2^.

### 2.5. Chemical–Physical Characterization

The morphology of the materials was studied by scanning electron microscopy (SEM). High-magnification microphotographs were performed by using an AURIGA, CrossBeam Workstation dual column Focused Ion Beam—SEM (Zeiss, Oberkochen, Germany) and a Jeol JSM-5510LV, both with an Everhart Thornley Detector (Jeol, Tokyo, Japan). The specimens were directly mounted onto a conductive double-face carbon tape, which was previously mounted on a slab. The surface chemistry was mapped with an X-ray energy dispersive spectroscopy (EDS) system (IXRF EDS-2000, Micro Pioneer, Seoul, Korea). The crystal structure of the material was characterized by X-ray powder diffraction (XRD) analysis (Smart Lab, Rigaku) using Cu-Kα radiation. The thermal stabilities were investigated by the simultaneous use of a thermo gravimetric (TG) and differential thermal analysis (DTA) instrument (Q600 SDT, TA Instruments, New Castel, DE, USA) equipped with the Thermal Solution Software (Version 1.4, TA Instruments). The reference for the calibration of temperature was the nickel Curie point. Ceramic standard, furnished with instrument, permitted the mass calibration. An aluminum oxide with high grade of purity was used as a comparison material. The experiments were performed with open platinum crucibles (cross-section = 0.32 cm^2^) using 10–12 mg of samples. Starting from room temperature, the sample was heated with a gradient of 10 °C·min^−1^ up to 850 °C. The onset temperature was calculated by thermal analysis software (Version 2.5, Universal Analysis, TA Instruments) as the intersection between the extrapolated baseline and the tangent through the inflection point of the weight vs temperature curve.

### 2.6. Electrochemical Characterization

For the electrochemical characterization, circular electrodes with a diameter of 12 mm were punched from the tape. Their electrochemical features were tested in a two-electrode sodium cell in which sodium acted as both the counter and reference electrode. The cells were set up by sandwiching a Whatman glass microfiber filter (Grade GF/A), used as the separator, between the positive and the negative electrode. The cycling performance and cycle life of the cells were evaluated in 2032-type coin cells. A 1.0 M solution of NaClO_4_ dissolved in propylene carbonate was used as the electrolyte. The cycling tests were automatically carried out with a battery cycler (Maccor 4000). All experimental activities were performed at 20 °C in a dry room (R.H. < 0.1% at 20 °C).

## 3. Results and Discussion

### 3.1. Chemical–Physical Characterization of the Sn-Decorated RGO

**Figure 1:**
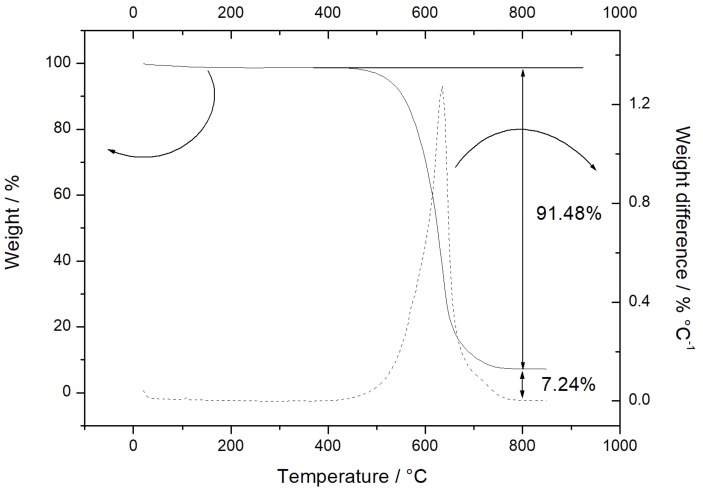
TGA curve (solid line) and weight difference (dash line) for the as-prepared Sn-RGO sample. The thermal analysis was conducted at a heating rate of 10 °C·min^−1^ from 20 to 850 °C.

**Figure 2:**
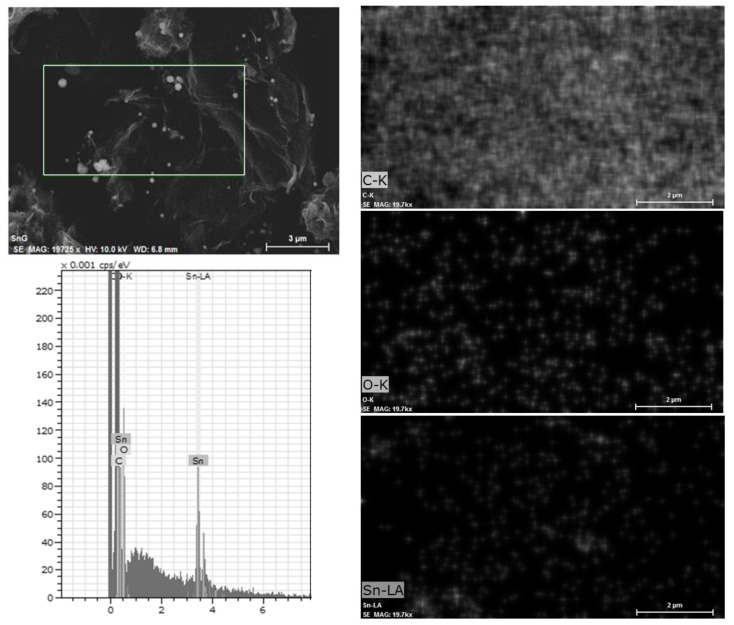
Upper left: Scanning electron microscopy (SEM) micrograph of the Sn-RGO; the rectangle bordered by the white line delimits the area where the EDS analysis was conducted. Lower left: EDS spectrum showing the presence of carbon, oxygen, and tin. Upper right: the white spots mark the presence of carbon. Center right: the white spots mark the presence of oxygen. Lower right: the white spots mark the presence of tin.

**Figure 3:**
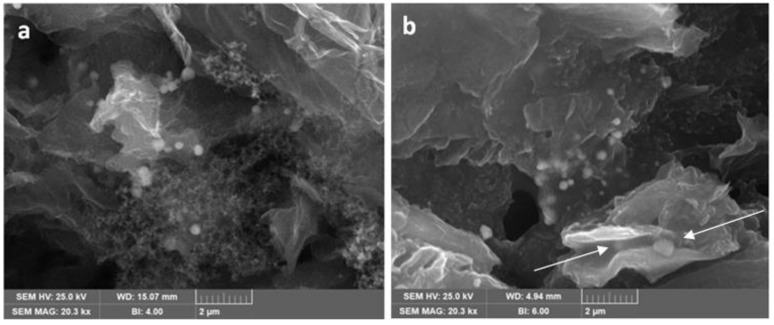
Scanning electron microscopy (SEM) micrograph of the Sn-RGO electrode before (**a**) and after (**b**) the electrochemical test.

The thermal properties of the Sn-RGO material and the percentage of tin and carbon were determined by TGA. The test was carried out in air at a heating rate of 10 °C·min^−1^ from 20 to 850 °C. The result of the test is reported in Figure 1. The Sn-RGO sample does not exhibit weight variations up to 400 °C, confirming the absence of labile oxygen-containing groups (alcoholic, carbonyl, or carboxylic). An important change in weight, corresponding to 91.48%, occurred between 550 and 650 °C. Such weight loss was associated with the oxidation of the graphene sheets present in the composite material. The DTA shows a single peak centered at 625 °C. The weight percentage of the final residue was about 7.24%. Since all the metal in the sample was converted into the corresponding oxide, this percentage represents the total tin content expressed as SnO_2_. Figure 2 (top left) shows the SEM image of the Sn-RGO. The graphene layers were observed as ripples at the bottom of the figure. The layers were partially restacked and wound together, with a structure resembling the exfoliate graphite obtained by heating the natural graphite. Spherical particles were deposited on the surface of the graphene sheets. The particles had a size between 200 and 400 nm and were highly dispersed and not homogeneously distributed on the graphene sheets. The EDS analysis was conducted on the rectangle delimited by the white line. The results are presented in Figure 2 (down left). The EDS allows identification of the presence of tin, carbon, and oxygen. The distribution maps of the three elements are depicted on the right of Figure 2. Since the main constituent of the sample was graphene, carbon was found uniformly distributed in all of the area under observation. Oxygen was uniformly dispersed in the graphene matrix. Tin was also dispersed in the graphene matrix but was also concentrated in correspondence of the spherical particle observed in the SEM image. The spherical particles were probably formed during the heating treatment at 800 °C by segregation of melted tin (the tin liquefaction point was about 232 °C). Figure 3 shows the SEM images related to the electrode material before (Figure 3a) and after (Figure 3b) the galvanostatic tests. The cell was tested for 10 cycles of charge–discharge and disassembled at the end of the last discharge process. In Figure 3a, it is possible to observe the spherical particles of Sn and also the presence of RGO sheets is detected. The image (Figure 3b) showed a more compact material, due to the fact that the surface roughness was reduced. This can be explained by the formation of an interphase between the electrode and the electrolyte caused by the partial degradation of the electrolyte itself. Moreover, the graphene sheets were stacked in multilayer structures with a thickness greater than 200 nm, but also observed was the presence of Sn particles (white arrows) between the graphene sheets, which limited their complete stacking.

**Figure 4:**
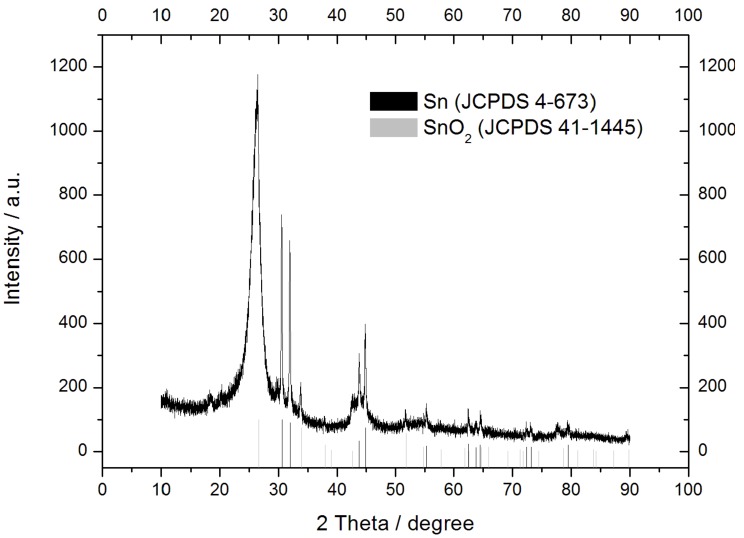
X-ray diffraction (XRD) patterns of the Sn-RGO. Black and gray lines represent the position and intensity of the tin and tin oxide diffraction peaks, respectively.

The XRD analysis (Figure 4) gives us information on the crystal structure of the tin present in the material. The peaks shown in Figure 4 can be indexed as belonging to metallic tin with tetragonal structure with space group 141:I4_1_/amd (Joint Committee on Powder Diffraction Standards (JCPDS) no. 4-673) and tin oxide with Cassiterite structure with space group 136:P4_2_/mnm (JCPDS no. 41-1445). The broad peak at 2θ = 26.42° may be attributed to disordered graphene nanosheets [46] partially restacked during the high temperature treatment.

### 3.2. Chemical–Physical Characterization of the Na_1.0_Li_0.2_Ni_0.25_Mn_0.75_O_δ_

**Figure 5:**
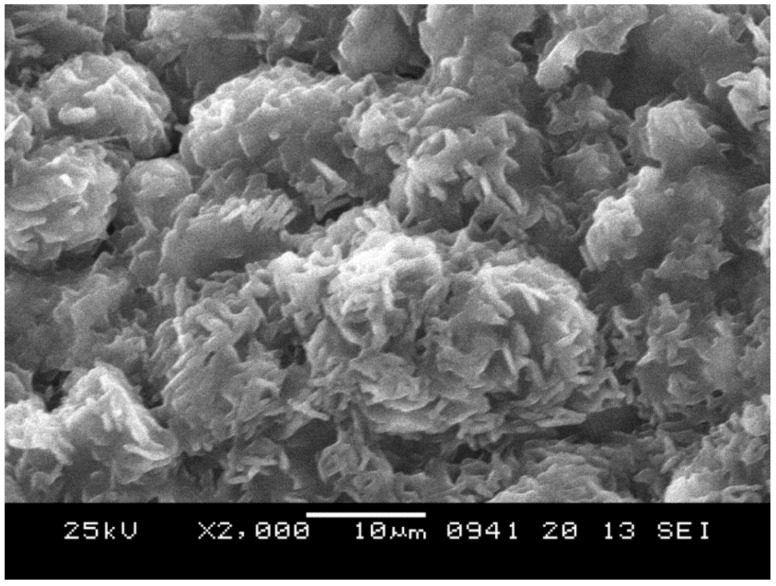
Scanning electron microscopy (SEM) micrograph of the Na_1.0_Li_0.2_Ni_0.25_Mn_0.75_O_δ_.

**Figure 6:**
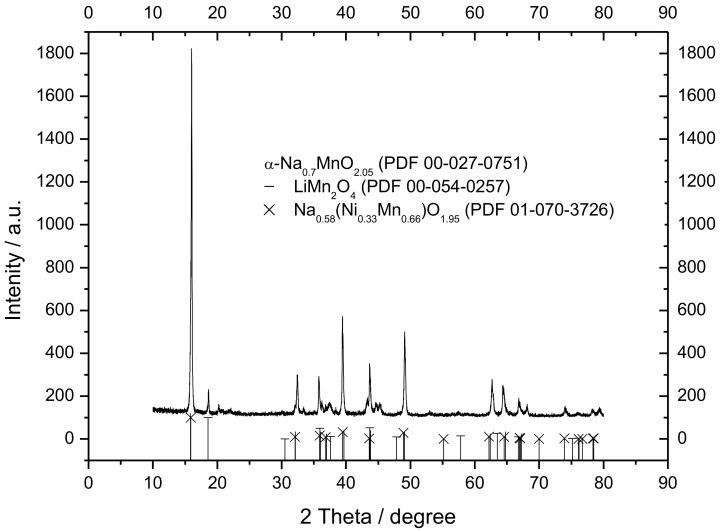
X-ray diffraction (XRD) patterns of the Na_1.0_Li_0.2_Ni_0.25_Mn_0.75_O_δ_. Black lines represent the position and intensity of the hexagonal P2-structure, space group P6_3_/mmc, of α-Na_0.70_MnO_2.05_.

Figure 5 shows an SEM micrograph of the Na_1.0_Li_0.2_Ni_0.25_Mn_0.75_O_δ_. It was possible to observe a compact platelet morphology with delaminated layers. This morphology should be attributed to the high synthesis temperature that allows grain growth and porosity reduction. The material appears formed by thin, rectangular primary structures with very jagged edges with length in the range of 3–10 μm. These primary structures were stacked in a disorderly manner. The result was the formation of a secondary structure with rippled and rounded edges, resembling a rose of the desert. The XRD of the material is shown in Figure 6. The reflection peaks were sharp and narrow, thereby indicating a high degree of crystallization. The peaks can be indexed according to the hexagonal P2-structure, space group P6_3_/mmc of α-Na_0.70_MnO_2.05_ (JCPDS no. 27-0751). Other small peaks can be attributed to secondary phases [47] LiMn_2_O_4_ and Na_0.58_Ni_0.33_Mn_0.66_O_1.95_.

### 3.3. Electrochemical Characterization of the Sn-Decorated RGO Electrode

**Figure 7:**
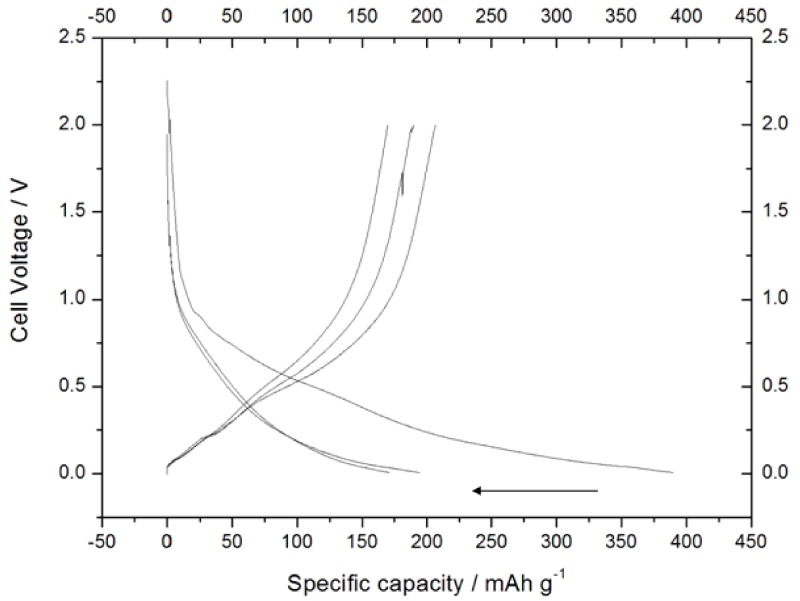
Voltage profiles recorded during the first three charge/discharge cycles for the Sn-RGO-based electrode. The cycles were conducted at 60 mA·g^−1^ between 0.05 V and 2.00 V. The arrow indicates the progression of the cycles.

**Figure 8:**
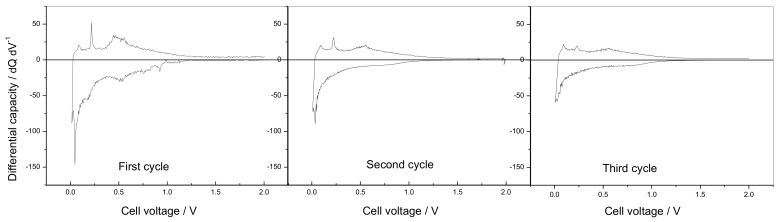
Differential capacity recorded during the first three cycles for the Sn-RGO-based electrode.

**Figure 9:**
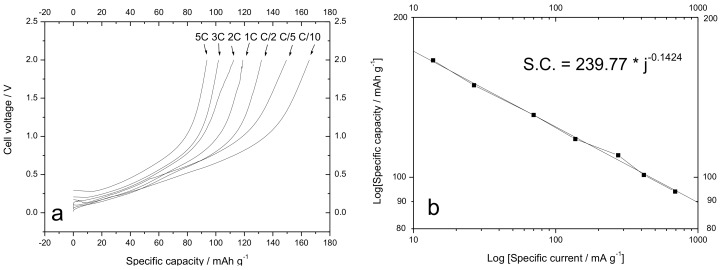
(**a**) Voltage profiles for the Sn-RGO-based electrode recorded at various charge rates as a function of the specific capacity. The charge rates are reported in the figure. (**b**) Log–log plot of the specific capacity as a function of the specific current. The equation inside the graph shows the result of the linear interpolation.

**Figure 10:**
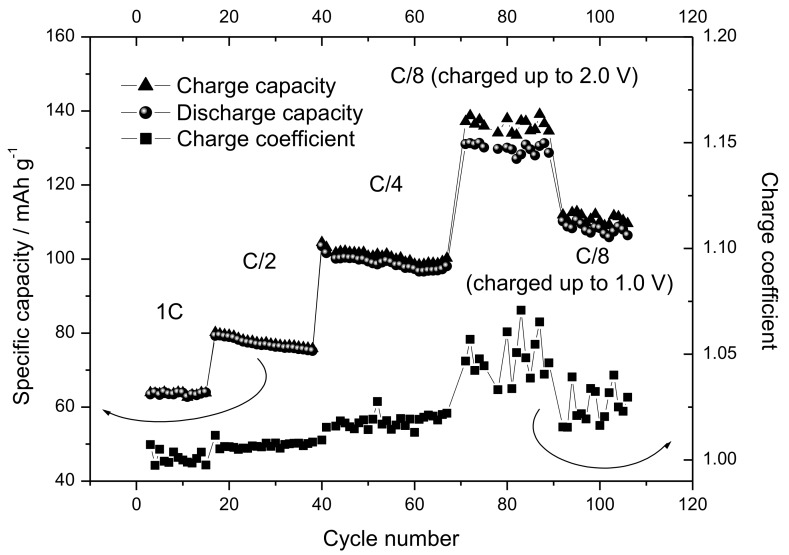
Specific capacity in charge (triangles) and discharge (circles) for the Sn-RGO-based electrode recorded at various charge rates as a function of the cycle number. The charge rates are reported in the figure. The squares indicate the charge coefficient.

The Sn-RGO electrode was first characterized by slow galvanostatic charge–discharge cycles. The Sn-RGO was used as the working electrode in a sodium cell in which sodium acted as the counter and the reference electrode. The cycles were conducted at 60 mA·g^−1^ (0.016 mA) between 0.05 V and 2.00 V and Figure 7 reports the corresponding voltage profiles. The discharge was completed exactly in 10 hours; for this reason, the C rate was calculated to be 0.16 mA. The first discharge cycle was characterized by a large irreversible capacity. In discharge, the cell was able to accommodate about 390 mA·h·g^−1^ (this specific capacity referred to the total weight of the electrode (Sn-RGO + carbon + Pattex PL50) without considering the weight of the current collector). During the following charge, the recovered capacity was about 205 mA·h·g^−1^, corresponding to an initial Coulombic efficiency of 52.5%. In the following charge cycle, the capacity was less than 200 mA·h·g^−1^ and about 97% was recovered in charge. At the third cycle, the cell began to work reversibly: the capacity was 170 mA·h·g^−1^ with a Coulombic efficiency of 100%. This capacity was assumed as the rated capacity of the electrode and all the rates used in the following work referred to this capacity. The source of the first and second cycles’ irreversible capacity can be ascribed to several factors: (i) the irreversible reduction of tin oxide [48], (ii) the trap of sodium-ion on the graphene sheets or unrepaired defect sites [49], and (iii) the formation of a stable solid–electrolyte interphase (SEI) layer [50]. To better understand what happens during the charge/discharge cycles, Figure 8 reports the dQ dE^−1^ vs. E differential capacity plot recorded during the first three cycles. In the first discharge cycle, the peak located at approximately 1.0 V was associated with the irreversible conversion reaction of SnO_2_ with sodium ion and the formation of SEI induced by electrolyte decomposition on the surface of the Sn-RGO. This peak disappeared during the subsequent cycle because the electrolyte decomposition was hindered due to the stable SEI formed on the electrode surface. The peaks at 0.18 V and 0.02 V can be ascribed to the alloying reaction between Sn and Na^+^ to form Na_x_Sn alloy. In the first charge step, two peaks at 0.075 and 0.22 V can be attributed to the dealloying of Na_x_Sn alloy. A broad anodic peak at 0.45 V corresponds to the capacitive storage behavior attributed to electroadsorption/desorption of sodium ions in RGOs [51,52]. By observing the second and third discharge cycles, it follows that absorption of sodium ions into the partially restacked graphene layer starts from 1.25 V and continues at lower voltage. In charge, the peak at 0.087 V remained unchanged while the peaks at 0.22 and 0.50 V decreased with the progression of the cycles. As a result, it was possible to conclude that both the tin alloying/dealloying mechanism and the chemisorption on heteroatoms and reversible surface physical adsorption on RGO contribute to the capacity of the Sn-RGO electrode. Figure 9a shows the voltage profiles of the Sn-RGO electrode when cycled at various charge rates. The electrode was always discharged at the current, corresponding to C/10 rate. At the lower charge rate, the electrode was able to deliver almost the rated capacity (165 mA·h·g^−1^). By increasing the charge current, a decrease in the capacity and an increase in the average charge voltage were observed. The capacity decreased down to 118 mA·h·g^−1^ at 1 C rate (about 70% of the rated capacity) and to 93 mA·h·g^−1^ at 5 C rate (about 38% of the rated capacity). The average cell voltage increased from 0.63 V (at C/10 rate) to 0.90 V (at C rate) and to 1.11 V at 5 C rate. Considering that the amount of tin inside the sample, in the form of Sn or SnO_2_, is very small (under 6%), the relative electrochemical contribution will be equally small. Consequently, the RGO contributes almost completely to the total capacity. As reported in the literature, the curves show the typical trend of the RGO [49]. With respect to pure RGO, the Sn-RGO presents a lower value of the first cycle’s irreversible capacity, a little bit higher capacity at low discharge rate, and a comparable rate capability. Figure 9b is a log–log plot in which the specific capacity was reported as a function of the specific current. A linear behavior was observed: from the linear regression it was possible to evaluate the power law that relates the specific capacity to the discharge current: S.C. = 239.77 × j^−0.133^ where S.C. is the specific capacity and j is the specific current. The capacity retention as a function of the cycle number is reported in Figure 10, together with the charge coefficient, namely the ratio between the amount of charge used to fully recharge the battery and the drawn charge. The cell was cycled in charge and discharge at the rates reported in the figure. Two aspects can be immediately noticed: (i) at the same rate, the specific capacity was lower with respect to the capacity observed in the previous experiment, and (ii) by decreasing the rate, the charge coefficient increases. The first aspect was easily explained considering that in the previous experiment, the cell was always discharged at the lower rate (C/10). In this experiment, the cell was charged and discharged at the same rate. It follows that the discharge conditions strongly influence the capacity of the electrode. The second aspect was more difficult to justify. A possible explanation could be the presence of a parasitic reaction, namely the oxidation of the electrolyte. This parasitic reaction, consuming part of the charge, could increase the charge coefficient. This reaction probably occurs at high voltage, in proximity of the end of the charge. At high charge rate, the period of time that the electrode remained at high voltage was low and the entity of the parasitic reaction was minimal. By reducing the discharge rate, the time that the electrode spent at high voltage increased and the contribution of the parasitic reaction rose. This behavior suggests that the source of parasitic reactions was unlikely rooted in irreversibility of the active Sn phase, but instead on some interfacial instability. In fact, different than for the Li-ion counterpart, the SEI formed in Na-ion batteries is known to be much thinner [50], mainly based on inorganic components and, above all, affected by instability both on carbon- and tin-based electrodes [53,54]. To validate the supposition that the irreversible processes mainly occur at high voltages, the end charge voltage of the electrode was lowered from 2.0 V down to 1.0 V maintaining the same charge rate (cycles from 90 to 106 in Figure 10). Correspondingly, a decrease of the charge coefficient was observed, confirming the validity of the supposition. It can thus be inferred that, in order to avoid battery failure, it is preferable to keep the anode voltage as low as possible to guarantee, at the same time, capacity and reversibility.

### 3.4. Electrochemical Characterization of the Cathode

**Figure 11:**
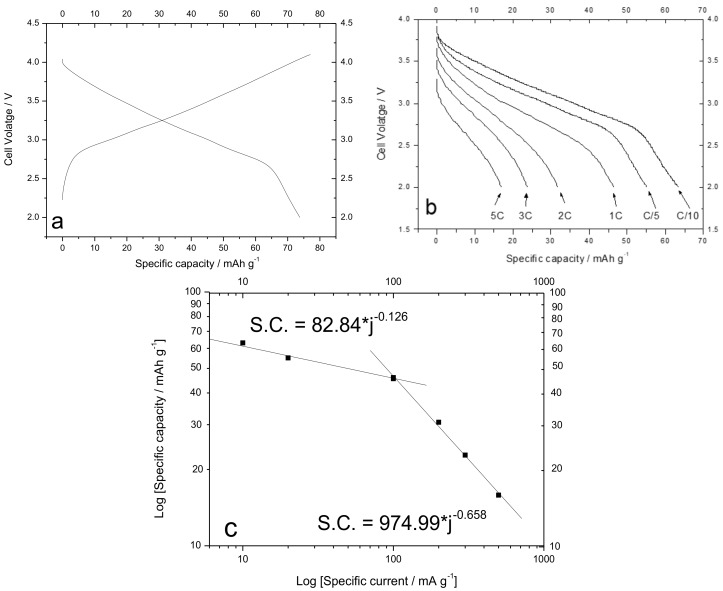
(**a**) Voltage profiles in charge and discharge for the Na_1.0_Li_0.2_Ni_0.25_Mn_0.75_O_δ_ electrode recorded C/10. (**b**) Discharge voltage profiles for the electrode at various rates as a function of the specific capacity. The discharge rates are reported in the figure. (**c**) Log–log plot of the specific capacity as a function of the specific current. The equation inside the graph shows the result of the linear interpolation.

Before assembling the battery, the cathode material (Na_1.0_Li_0.2_Ni_0.25_Mn_0.75_O_δ_) was tested in a sodium half-cell to evaluate its electrochemical properties. The cell was cycled at C/10 rate (0.1 mA) and Figure 11a reports the voltage profiles in charge and discharge. In charge, the voltage rapidly increased from the end charge limit (2.0 V) up to less than 3.0 V. After that, the voltage linearly increased with time. The same behavior was observed in discharge. The specific capacity in charge (about 77 mA·h·g^−1^) was a little bit higher than the capacity in discharge (about 74 mA·h·g^−1^) with a Coulombic efficiency of about 96%. The average discharge voltage was 3.14 V. With respect to the analogous material synthesized by Kim et al. [40], this material presents a lower specific capacity at low discharge rate, a lower rate capability, and a lower average discharge voltage. The low value of the specific capacity and the low average discharge voltage were disadvantageous for the production of high-energy density batteries. The energy density of the material (about 240 W·h·kg^−1^) was about one half of the energy density of the materials usually used in LIB technology. Furthermore, in some cases, a parasitic reaction affected the electrode. The parasitic reaction, most likely associated with the oxidation of the electrolyte, avoided the cell to reach the end charge voltage. The appearance of the parasitic reaction was random as some cells had worked for several cycles before the parasitic reaction was established. Instead, in other cases the parasitic reaction started already after the first cycle. This behavior has been repeated in subsequent cycles and also in other cells, confirming a substantial instability of the electrolyte to work with this cathode material. Once again, these findings agree with a more pronounced interfacial instability for SIBs than for LIBs, mainly because of the higher solubility of electrolyte decomposition products (e.g., Na_2_CO_3_, which was the main interphase component together with NaPF_6_) in organic electrolytes with respect to the Li counterparts [55]. Figure 11a shows the voltage profiles of the Na_1.0_Li_0.2_Ni_0.25_Mn_0.75_O_δ_ electrode when cycled at various discharge rates. The electrode was always charged at the lower discharge current (0.01 mA), corresponding to the C/10 rate. At the lower discharge rate, the capacity was a little bit lower than the previously rated capacity (63 mA·h·g^−1^). By increasing the discharge current, the capacity decreased down to 50 mA·h·g^−1^ at 1 C rate and to less than 20 mA·h·g^−1^ at 5 C rate. The average cell voltage decreased from 2.8 V (at 1 C rate) to 2.6 V (at 5 C rate). Figure 8 reports in a log–log scale the specific capacity as a function of the specific current. At specific currents lower than 100 mA·g^−1^, the loss of capacity with increasing current was quite low: from the linear regression, the power low that relates the specific capacity to the discharge current was S.C. = 82.84 × j^−0.126^. For higher specific currents, a more severe capacity fading was observed: the pre-exponential factor increased while the exponential factor decreased and the power low assumed the form: S.C. = 974.99 × j^−0.658^.

### 3.5. Electrochemical Characterization of the SIB

**Figure 12:**
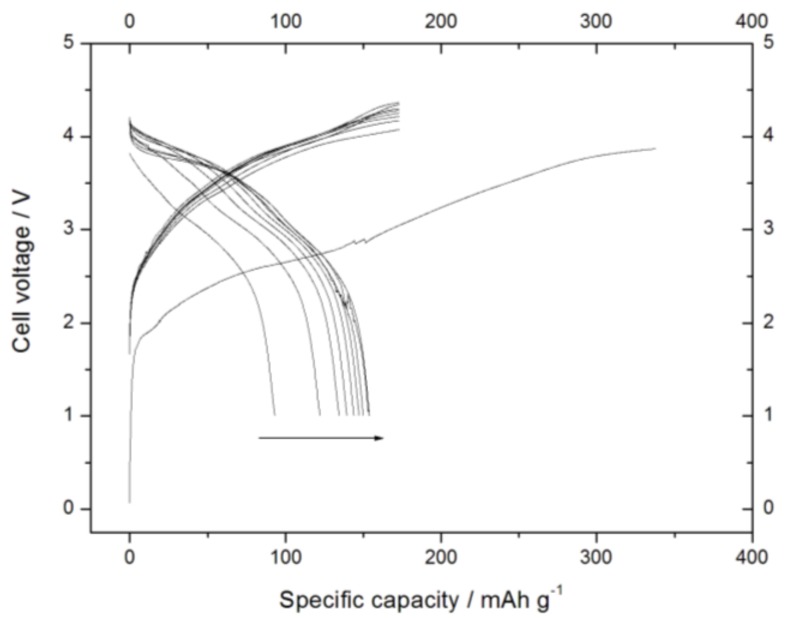
Voltage profiles recorded during the first 10 charge/discharge cycles for the Sn-RGO/Na_1.0_Li_0.2_Ni_0.25_Mn_0.75_O_δ_ SIB. The cycles were conducted at 0.088 mA between 4.4 V and 1.0 V. The arrow indicates the progression of the cycles.

**Figure 13:**
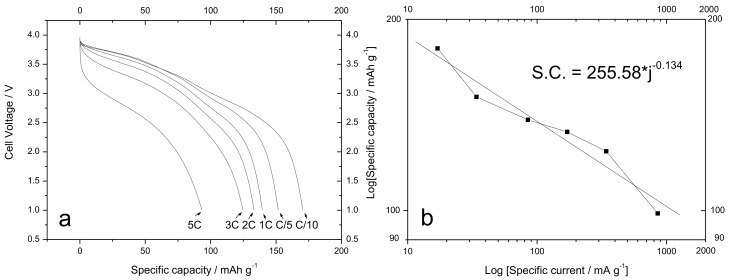
(**a**) Voltage profiles for the Sn-RGO/Na_1.0_Li_0.2_Ni_0.25_Mn_0.75_O_δ_ SIB recorded at various discharge rates as a function of the specific capacity. The charge rates are reported in the figure. (**b**) Log–log plot of the specific capacity as a function of the specific current. The equation inside the graph shows the result of the linear interpolation.

**Figure 14:**
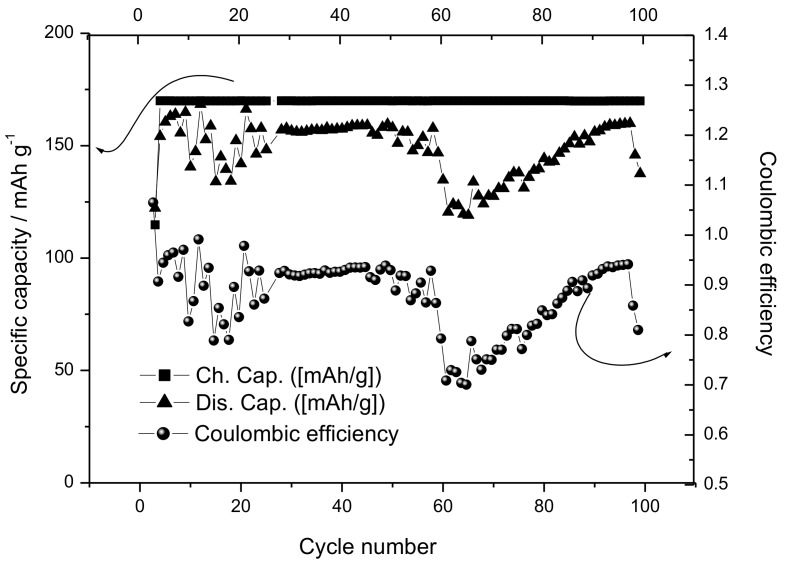
Specific capacity as a function of the cycle number for the Sn-RGO/Na_1.0_Li_0.2_Ni_0.25_Mn_0.75_O_δ_ SIB.

Despite the possible occurrence of the parasitic process, which could profoundly affect the result of the test, a SIB was prepared using the Sn-RGO as the anode and the Na_1.0_Li_0.2_Ni_0.25_Mn_0.75_O_δ_ as the cathode. The weight of the anode active material was 5.2 mg. Considering a reversible capacity of 170 mA·h·g^−1^, the capacity of the electrode was 0.88 mA·h. The weight of the cathode active material was several times larger than the anode (about 26 mg). Considering a reversible capacity of 74 mA·h·g^−1^, the capacity of the cathode was about two times the capacity of the negative electrode. The cell was cycled between 4.4 V and 1.0 V at a current of 0.088 mA (C/10 rate). A time limit of 20 h was set for the first charge step and of 10 h for the following charge steps. Figure 12 shows the voltage profiles recorded during the test. In the first charge, the cell reached the time limit (20 h) charging about 380 mA·h·g^−1^. During the following step, only 93 mA·h·g^−1^ were discharged. In the following charges, the cell always reached the second time limit (10 h) charging 170 mA·h·g^−1^ (the rated capacity of the anode). In discharge, the capacity slowly increased, reaching about 154 mA·h·g^−1^ after 10 cycles. From this evidence, it follows that the irreversible process does not run out during the first cycles but it affects the cell throughout its operation. Among the various possible causes of this behavior, the formation of sodium metal on the surface of the negative electrode cannot be ruled out, with a consequent decrease in Coulombic efficiency, but this aspect deserves to be further clarified. Figure 13a shows the voltage profiles for the previously described SIB when cycled at various discharge currents (C/10, C/5, 1 C, 2 C, 3 C, and 5 C). The charge was carried out at the same current rate (C/10 rate). At the lower discharge rate, the cell capacity approached the rated value (170 mA·h·g^−1^). By increasing the discharge current, the capacity decreased but the decline was not so pronounced: the cell discharged about 80% of the rated capacity at 1 C rate and more than 50% at 5 C rate. Figure 13b reassumes the result obtained in the previous experiment: the specific capacity was plotted as a function of the specific current in a log–log plot. Also, in this case, it was possible to observe that the capacity decreased linearly with the increase of current. From the linear fit, the pre-exponential factor was found to be 255 while the exponential factor of the specific current was found to be 0.134, lower than the value found for the cathode when cycled at high discharge currents. From this evidence, it is possible to state the SIB was not kinetically limited by the cathode since, due to the large amount of cathode material into the cell, the discharge current did not exceed the cathode limiting current. Figure 14 shows the charge–discharge cycling test of the SIB. The weight of the anode was 2.3 mg. Considering a reversible capacity of 170 mA·h·g^−1^, the capacity of the electrode was 0.4 mA·h. Also, in this case, the SIB was anode-limited: the cathode capacity was at least three times larger. The cell was cycled between 4.4 V and 2.0 V at a current of 0.4 mA (1 C rate) in charge and 0.2 mA (C/2 in discharge). A time limit of 1 h was set in charge. The cell never reached the end charge voltage but the charge was stopped after 1 h, storing the practical capacity (170 mA·h·g^−1^). In discharge, the cell capacity increased with the cycle number to reach a stable value of about 160 mA·h·g^−1^ after seven cycles, reflecting a possible anode activation upon cycling probably due to progressive wetting by electrolyte and involvement of surface charge storage domains. In following cycles, the discharge capacity was not constant and the Coulombic efficiency was found to change very suddenly. This behavior can be explained by considering the concomitant presence of an irreversible process in charge, the extent of which randomly varies with the number of cycle. This irreversibility can be ascribed to the low interfacial stability of electrolyte with the cathode. As a consequence, the capacity in discharge was variable, depending on the entity of the irreversible process in the previous charge cycle. Nevertheless, the cell cycled for over 100 cycles with capacities ranging from 120 up to 160 mA·h·g^−1^.

## 4. Conclusions

A Sn-decorated RGO, originally designed to be used as an electrode for lithium-ion batteries, was tested as the anode active material for SIB. The Sn-RGO showed a high irreversible first cycle capacity, but in the following cycle, a stable SEI layer was developed and the cell began to work reversibly. It was found that both the tin alloying/dealloying and the chemisorption on heteroatoms on RGO contribute to the reversible capacity of the electrode, and that the anode discloses an appreciable response in power. At low charge/discharge rates, an anomalous high value of the charge coefficient presupposes the existence of an electrolyte parasitic reaction that occurs at high voltage, close to the end of the charge.

Sodium-based layered oxide with the formula Na_1.0_Li_0.2_Ni_0.25_Mn_0.75_O_δ_ was synthesized, characterized, and electrochemically tested as a cathode active material in a sodium cell. The discharge-specific capacity increased with cycling. A parasitic reaction, namely the oxidation of the electrolyte, was observed to occur randomly upon charge.

To evaluate the effectiveness of the Sn-RGO electrode as the anode of an SIB, it was tested coupled with Na_1.0_Li_0.2_Ni_0.25_Mn_0.75_O_δ_ cathode. The electrochemical characterization allowed the confirmation that the battery was able to reversibly cycle sodium ions. The cell’s power response was fairly good and despite the presence of the irreversible process in charge, the cell cycled for over 100 cycles with capacities ranging from 120 up to 160 mA·h·g^−1^.
